# Biofilm cultivation facilitates coexistence and adaptive evolution in an industrial bacterial community

**DOI:** 10.1038/s41522-022-00323-x

**Published:** 2022-07-20

**Authors:** Nathalie N. S. E. Henriksen, Mads Frederik Hansen, Heiko T. Kiesewalter, Jakob Russel, Joseph Nesme, Kevin R. Foster, Birte Svensson, Gunnar Øregaard, Jakob Herschend, Mette Burmølle

**Affiliations:** 1 Section of Microbiology, Department of Biology, University of Copenhagen, Universitetsparken 15, 2100 Copenhagen, Denmark; 2 Enzyme and Protein Chemistry, Department of Biotechnology and Biomedicine, Technical University of Denmark, 2800 Lyngby, Denmark; 3 Department of Zoology, University of Oxford, Oxford, UK; 4 Department of Biochemistry, University of Oxford, Oxford, UK; 5 Discovery, Research and Development, Chr. Hansen A/S, 2970 Hørsholm, Denmark; 6 Present Address: Section for Microbial and Chemical Ecology, Department of Biotechnology and Biomedicine, Technical University of Denmark, 2800 Lyngby, Denmark; 7 Present Address: Functional Food, Novozymes A/S, 2800 Lyngby, Denmark

**Keywords:** Biofilms, Evolution, Microbial communities, Industrial microbiology

## Abstract

The majority of ecological, industrial and medical impacts of bacteria result from diverse communities containing multiple species. This diversity presents a significant challenge as co-cultivation of multiple bacterial species frequently leads to species being outcompeted and, with this, the possibility to manipulate, evolve and improve bacterial communities is lost. Ecological theory predicts that a solution to this problem will be to grow species in structured environments, which reduces the likelihood of competitive exclusion. Here, we explored the ability of cultivation in a structured environment to facilitate coexistence, evolution, and adaptation in an industrially important community: *Lactococcus lactis* and *Leuconostoc mesenteroides* frequently used as dairy starter cultures. As commonly occurs, passaging of these two species together in a liquid culture model led to the loss of one species in 6 of 20 lineages (30%). By contrast, when we co-cultured the two species as biofilms on beads, a stable coexistence was observed in all lineages studied for over 100 generations. Moreover, we show that the co-culture drove evolution of new high-yield variants, which compared to the ancestor grew more slowly, yielded more cells and had enhanced capability of biofilm formation. Importantly, we also show that these high-yield biofilm strains did not evolve when each species was passaged in monoculture in the biofilm model. Therefore, both co-culture and the biofilm model were conditional for these high-yield strains to evolve. Our study underlines the power of ecological thinking—namely, the importance of structured environments for coexistence—to facilitate cultivation, evolution, and adaptation of industrially important bacterial communities.

## Introduction

Microorganisms commonly reside in communities where interspecies interactions govern the ecological function. For example, some microbes depend on the metabolic waste products of other species^1^, some are antagonized by competing species^2^ and some gain synergistic community-intrinsic protection from stresses^3^. The community structure is often complex and challenging to study. A common observation under homogeneous conditions is that species diversity is rapidly reduced due to competitive exclusion^4–6^. Coexistence requires niche differentiation that increases intraspecific competition, so it becomes stronger than interspecific competition^7^. Introduction of a structured environment can generate distinct niches, and hence lead to coexistence and increased diversity^5,6,8^.

Here, we study the coexistence and evolution of bacteria in a biofilm-selecting environment. In biofilms, cells are encased in an extracellular matrix that ensures heterogeneity, structural rigidity, spatial partitioning and facilitates coexistence between strains that occupy different micro niches^9–11^. The continuous presence of multiple species impacts evolution^12–14^ and hence we hypothesized that the dual-species biofilm would generate and maintain unique variants. To study this, we used the routinely applied starter culture species *Leuconostoc mesenteroides* and *Lactococcus lactis* as a model system. Lactic acid bacteria are interesting in this context as they represent a community with a high level of interspecies dependency where each species concomitantly contribute to the production of dairy products^15^. This model is also relevant as the food industry may benefit from experimental evolution to improve bacterial properties^16^.

## Main text

**Fig. 1:**
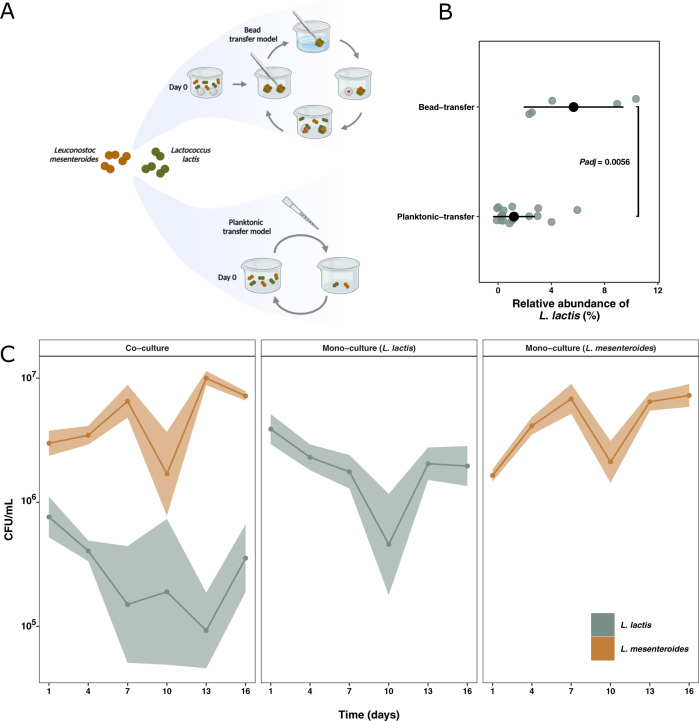
The modified bead-transfer model procedure and culture dynamics of *Lactococcus lactis* and *Leuconostoc mesenteroides* during 16 days of cultivation. **A** In the bead transfer model, *L. lactis* and *L. mesenteroides* were cultured individually or together on glass beads. After 24 h of cultivation, a bead with attached biofilm was washed three times and moved to a new container with labeled glass beads (marked with dots). Bacterial cells were allowed to disperse and attach to the new labeled beads for 24 h before transfer. At every third transfer, a single bead was sonicated to release and disperse biofilm cells for CFU quantification and isolation of evolved strains (Illustration created with Biorender.com). **B** Relative abundance of *L. lactis* in co-cultures with *L. mesenteroides* at day 16 in the bead-transfer model and planktonic-transfer model. The relative abundance of *L. lactis* was significantly higher in the bead-transfer model at day 16 compared to planktonic-transfer (*P* = 0.0056, Wilcoxon Rank-Sum test). **C** Cell counts of *L. lactis* and *L. mesenteroides* for mono- and co-cultures throughout the duration of the bead transfer experiment. Numbers of *L. mesenteroides* were unaffected by the co-cultivation (*P* > 0.05, Welch Two Sample *t* test), except for day 1 and 13 (*P* = 0.004 for both, Welch Two Sample *t* test), when comparing mono- and co-culture populations, whereas the number of *L. lactis* were reduced by co-cultivation compared to mono-cultivation (*P* < 0.05, Welch Two Sample *t* test, except day 10 where *P* = 0.33). Graph depicts means and 95% confidence intervals as solid lines and ribbons, respectively. Five biological replicates (lineages) were included. *N* = 5.

**Fig. 2:**
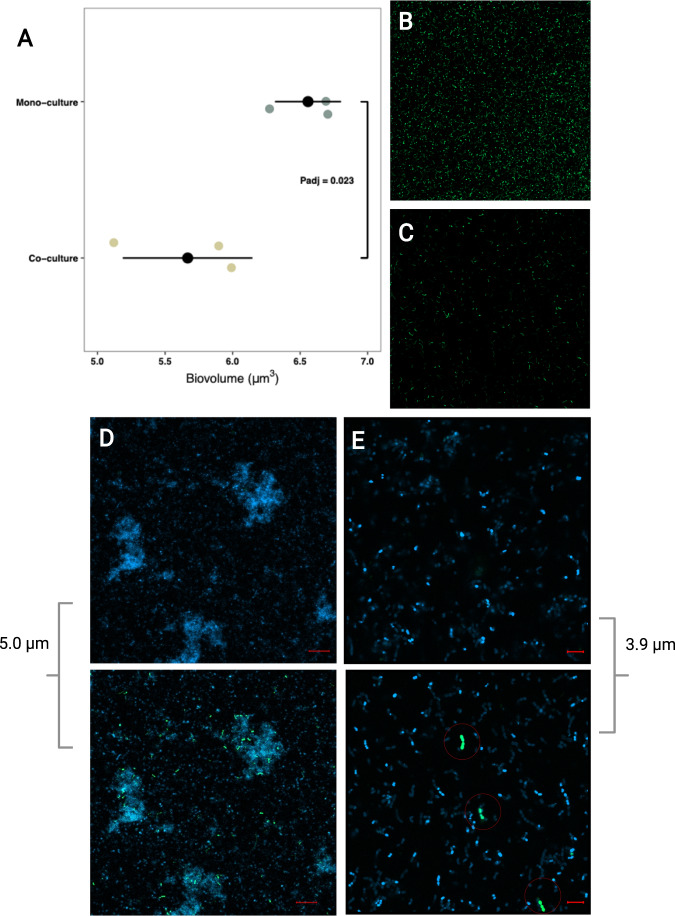
CLSM quantification of *Lactococcus lactis* abundance and localization on glass slides. **A** Numbers of *L. lactis* harboring pNAMA_P32-sfgfp(Bs)-CmR were lower when co-cultivated with *L. mesenteroides* on a glass surface compared to those of the single culture (*P*_adj_ = 0.023, Welch Two Sample *t* test, *N* = 3). Quantification of biovolume was performed using RCon3D and all replicates were imaged in a fixed volume of space (*X*:*Y*:*Z* 319.45 µm:319.45 µm:12 µm). The x-axis shows the log10 transformed biovolume. **B**, **C** Representative CLSM images of *sfGFP*(Bs)-expressing *L. lactis* incubated in mono culture and in co-culture with *L. mesenteroides*, respectively. **D** Visualization of DAPI-stained co-culture of *L. mesenteroides* and *sfGFP*(Bs)-expressing *L. lactis* confirmed that *L. lactis* was only present at the bottom in close proximity to the glass surface, while *L. mesenteroides* was still present 5 µm above (×20 magnification, scale bar represents 20 µm. Bottom image was acquired near the glass surface, top image is 5 µm above). **E** The observation in **D** was also evident with higher magnification (×63 magnification, scale bar represents 5 µm. Bottom image was acquired near the glass surface, top image is 3.9 µm above). Red circles in bottom image indicate *L. lactis* cells observed near the glass surface.

Initially we tested whether a biofilm-selecting environment minimized the loss of diversity i.e., enabled continuous presence of both species. As a biofilm-selecting environment we modified the bead-transfer-model originally developed by Poltak and Cooper^17^ (Fig. 1A). After a 16 days period with daily transfers (800-fold dilution, corresponding to bead transfer dilution (Supplementary Table 1)), we found that *L. lactis* was completely outcompeted in 6 out of 20 lineages in liquid culture (Fig. 1B). Conversely, the addition of a bead surface, onto which cells could adhere, ensured coexistence in all lineages and significantly higher relative abundance of *L. lactis* (Wilcoxon Rank-Sum test, *P* = 0.0056) (Fig. 1B). This observation emphasizes that spatial structure tends to reduce the ability of one species excluding the other, likely because the biofilm provides separate ecological niches that these strains occupy when they are otherwise competitors in liquid culture. To test whether the dominance of *L. mesenteroides* could be explained by higher tolerance to acidic conditions, we measured the pH of the liquid growth medium of the two species grown individually and in combination. The acidification of the spent medium of both species and the co-culture was however comparable and did not suggest that a co-culture environment would be unfavorable for *L. lactis* in terms of pH (Supplementary Fig. 1A). Interestingly, temporal quantification of cells on beads revealed that only the abundance of *L. lactis* was affected by the presence of another species (*L. lactis*: Welch Two Sample *t* test, *P* < 0.05 all days, except day 10) (*L. mesenteroides*: *P* > 0.05, Welch Two Sample *t* test, except for day 1 and 13) (Fig. 1C). To verify the quantification, a reporter plasmid encoding *sfGFP* was constructed to visualize *L. lactis* cells in situ on glass. Acquisition of confocal laser scanning microscopy images confirmed that the biomass volumes of fluorescent *L. lactis* cells were significantly lower when *L. mesenteroides* was present (Welch Two Sample *t* test, *P* = 0.023) (Fig. 2A–C). In combination with the application of DAPI staining, it became evident that both species competed for attachment on the glass bead surface, and subsequently, *L. mesenteroides* attached onto *L. lactis,* while the opposite was not observed. *sfGFP*-expressing *L. lactis* was only present at the bottom layer, and when moving the focal point 4–5 µm higher up, only DAPI-stained *L. mesenteroides* was observed (Fig. 2D, E). This may explain why *L. mesenteroides* reached the same cell numbers in co- and mono-species cultures, while *L. lactis* did not.

During the experiments we identified two colony morphotypes of *L. lactis*. These were denoted L- and S-morphotype, respectively (Fig. S2A). The S-morphotype colonies were smaller and darker, and planktonically grown cells of this morphotype had a distinct tendency to entangle, clump and sink to the bottom (Fig. S3). Also, number of cells in chains differed between the two types, with the S-morphotype having approx. 3 cells more per chain in average (Fig. S2C). Both colony morphotypes emerged from planktonic and biofilm mono-species cultivation, but only under biofilm conditions in co-cultivation, not in planktonic co-cultivation (Fig. S2B). This highlights that biofilm formation maintains a high level of diversity and includes variants that are easier outcompeted in planktonic cultures among other species.

**Fig. 3:**
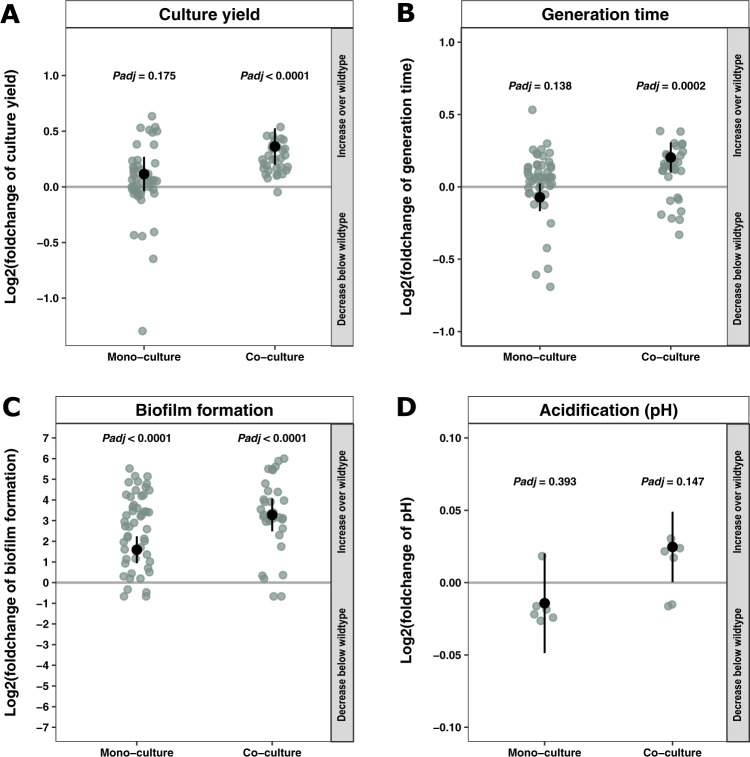
Co-cultivation increased culture yield, generation time, biofilm formation and final pH of *Lactococcus lactis* isolates. **A** Comparative analyses of *L. lactis* isolates emerging from mono- and co-cultivation, respectively, revealed that co-cultivation enabled unique evolution of isolates with significantly increased culture yield compared to the ancestor (*P*_adj_ < 0.0001, MELM). Culture yield of all mono- and co-culture isolates was normalized to the culture yield of the ancestor. **B** Comparison of generation times from growth-monitored cultures of ancestor, mono- and co-culture isolates of *L. lactis*, based on OD_600_ measurements and the Growthcurver package. The increased generation time was unique for the co-culture isolates (*P*_adj_ = 0.0002, MELM). Growth rates of all mono- and co-culture isolates were normalized to the generation time of the ancestor. **C** Comparison of biofilm formation under static conditions in the Calgary device using crystal violet to assess biofilm formation after 48 h of growth. Both mono- and co-culture isolates formed significantly more biofilm than the ancestor (*P*_adj_ < 0.0001, for both, MELM). **D** Comparison of final pH level in spent medium after 24 h of growth in a subset of isolates. No significant differences in pH development were detected in the tested isolates compared to the ancestor (*P*_adj_ = 0.393 and 0.147, MELM). Differences between mono- and co-culture isolates and the ancestor were assessed with a mixed-effects linear model. One observation represents the average of four biological replicates for culture yield and generation time (**A**, **B**, *N* = 79), while one observation represents the average of three biological replicates for biofilm formation and acidification (**C**, **D**, *N* = 85 and 12, respectively). Black symbols represent estimated population mean by MELM, error bars represent 95% confidence intervals and green dots represent observations. *P* values are adjusted using FDR.

To assess the evolutionary impact of coexistence, 83 evolved isolates of *L. lactis* were isolated from across the mono- and dual-species beads at day 14 (corresponding to approx. 100 generations (Supplementary Table 1)) and compared to the ancestor. The growth metrics, i.e., culture yield and generation time, were measured, computed, and tested using mixed-effects linear models (MELM). Culture yield was designated as the maximum optical density (OD_600_) reached during growth. We found that isolates originating from co-cultivation had significantly enhanced culture yield (*P*_adj_ < 0.0001, MELM; Fig. 3A) and generation time (*P*_adj_ = 0.0002, MELM; Fig. 3B) compared to the ancestor. This trend was not observed for isolates that emerged from mono-cultivation (*P*_adj_ = 0.175, MELM; Fig. 3A) (*P*_adj_ = 0.138; MELM; Fig. 3B). In addition, cell number quantification by qPCR at the maximum cell density verified that isolates emerging from co-cultivation were indeed able to reach a higher copy number than isolates originating from mono-cultures (*P*_adj_ < 0.0001, MELM; Supplementary Fig. 4). This verified that the increased culture yield in co-culture evolved isolates was in fact reflecting an increase in the number of cells and not an artifact caused by changes in cell size or composition.

To further decipher the impact of the cultivation method, phenotypic variables were included in the mixed-effects linear model with cultivation method and morphotype as variables. The type of cultivation (mono- vs. co-culture) was the only significant factor (*P*_adj_ = 0.007, MELM; Supplementary Figure 5A) for culture yield, while both culture type and morphotype impacted generation time (*P*_adj_ < 0.0001 and *P*_adj_ = 0.0004, MELM; Supplementary Fig. 5B).

Spatial constrictions have previously been found to favorite high-yield *L. lactis* strategists mutants, otherwise outcompeted in planktonic suspension^6^. Continued propagation of a chemically mutagenized population in the structured environment led to identification of a slow-growing mutant with high culture yield, with increasing prevalence over time^6^. Interestingly, in the present study, we identified a similar trade-off emerging in biofilms without the use of random mutagenesis. We did, however, only do so in *L. lactis* that had been co-cultivated with *L. mesenteroides* and not in cultures where this species was cultured on beads alone (Fig. 3A, B). This finding suggests that co-adaptations can accelerate the diversification of *L. lactis* into novel variants and that the biofilm environment provides these variants an advantage.

It has been suggested that the inherent design of the bead-transfer-model selects for a flexible “life-history” strategy, favoring combined abilities of rapid surface attachment, biofilm growth, dispersal, and then recolonization. In other words, strong biofilm specialists are only successful under short terms^18^. To test how the biofilm capabilities of *L. lactis* evolved and whether interspecies interactions impact this property, we quantified biofilm formation of the isolates. Both evolved mono- and co-culture isolates were significantly better at forming biofilm compared to the ancestor (*P*_adj_ < 0.0001 for both culture variants, MELM; Fig. 3C). However, a significant difference was also found between the two culture types, as the co-culture isolates were significantly better at biofilm formation than the mono-culture isolates (*P*_adj_ = 0.002, MELM; Supplementary Fig. S5C). In addition, a significant difference was also found between the two different colony morphotypes, explained by cells of the L-morphotype producing more biofilm than the S-morphotype (*P*_adj_ < 0.0001, MELM; Supplementary Fig. 5C). This was surprising as we expected the darker colonies of the S-morphotype on Congo red and Coomassie blue plates (Supplementary Fig. 2A) to be associated with enhanced expression of polysaccharides or proteins, which are commonly present in a biofilm matrix. It is however important to notice that the S-morphotype tended to sediment (Supplementary Figure 3), and hence its presence in the microwell liquid phase and encounters with the pegs extruding from the lids might be reduced compared to the ancestor and L-morphotype, which could lead to lower levels of biofilm formation in the PEG lid assay used for biofilm quantification.

As a final parameter, the acidification potential of the isolates was assessed, due to its relevance in terms of dairy production. Measurements of pH post 24 h of incubation did however not reveal a significant difference from the ancestor for neither mono- nor co-cultivation isolates (Fig. 3D).

**Fig. 4:**
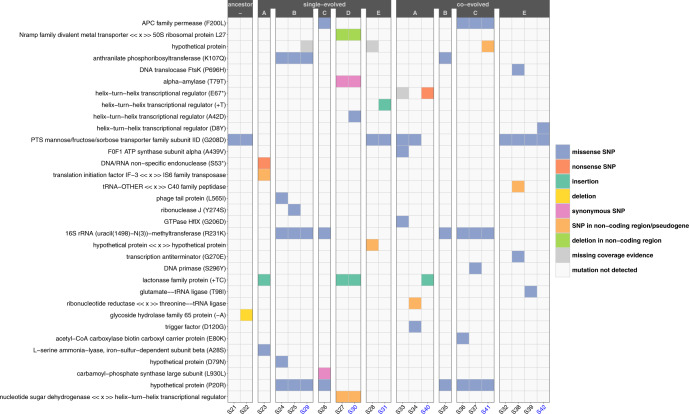
Predicted mutations in ancestral and evolved *Lactococcus lactis* isolates reveal distinct genotypes emerging from mono- and co-cultures. A selection of L-(black-labels) and S-morphotype (blue-labels) isolates (*X*-axis) evolved in mono-cultures or co-cultures, respectively, and two ancestors were selected for whole-genome sequencing. Predicted mutations by the computational pipeline breseq, including SNPs, insertions, and deletions in coding and non-coding regions, are arranged according to the isolates’ evolutionary background (ancestor, mono- or co-culture) and experimental lineage (A–E). *Y*-axis with gene descriptions is listed according to the chromosomal position. Some of the unique mutations identified for co-cultivation are associated with growth, for example an ATP synthase or DNA translocase. In contrast, unique mutations of mono-culture variants are associated with for example an endonuclease. Noticeably, mutations in a helix–turn–helix transcriptional regulator were only identified in the S-morphotype, which suggests that this morphotype is associated with a change in this regulator. For more detailed information on chromosomal position and mutations, see Supplementary Table 2.

To identify genomic differentiation, we sequenced 20 isolates, representing mono- and co-culture and the S- and L-morphotype from different lineages. Although we identified 31 unique mutations classified as single nucleotide polymorphisms (SNPs), insertions, and deletions of which 71% occurred in coding regions, we did not identify a specific gene that had mutated in most co-culture isolates (Fig. 4). However, it is relevant to note that this experiment was limited to approx. 100 generations (Supplementary Fig. 1) and with daily transfer representing a bottleneck. Hence, most mutations are lost by genetic drift and advantageous mutations thus require more generations to constitute a significant fraction of the population^19^. We did however identify an intriguing pattern; variants emerging from co-cultivation tended to have mutations in genes related to growth, such as DNA translocase or ATP synthase. Conversely, no such genetic modifications were identified in variants emerging from mono-cultures (Fig. 4 and Supplementary Table 2). Moreover, we identified mutations in a gene encoding a protein associated with a helix–turn–helix DNA-binding motifs in four of the six S- morphotype isolates. Such proteins often coordinate transcription of genes according to environmental conditions and act as regulators^20^. The remaining two S-morphotypes either had a SNP (S41 Fig. 4) or a potential SNP (S29, Supplementary Table 2) in a pseudogene upstream of this regulator. Thus, the S-morphotype is likely a result of mutations in this gene or regions that alter expression of this gene. Helix-turn-helix binding motifs are associated with 41 genes in the genome of this *L. lactis* strain, of which 22 are annotated as regulators and 4 as winged regulators. The specific gene of interest is relatively small, and the expressed protein only consists of 68 amino acids. When conducting a BLASTp^21^ sequence analysis, all matches with an identity higher than 85% were assigned to *Lactococcus* spp., indicating that this regulator could be specific for this genus.

In conclusion, our findings highlight that emergence and stabilization of a diverse and unique community are conditional on biofilm and interspecies interactions. Specifically, we show that a structured environment is a key determinant for coexistence and evolution. In a broader context, this study highlights the potential importance of eco-evolutionary thinking for microbial bioproduction^22^.

## Methods

### Bacterial strains and conditions

The ancestral strains, *L. lactis* subsp. *lactis* DSM-20481 and *L. mesenteroides* subsp. *mesenteroides* DSM-20343, were collected from Leibniz Institute DSMZ - German Collection of Microorganisms and Cell Cultures (DSMZ) (Braunschweig, Germany). Ancestral strains and emerging isolates (Supplementary Table 3) were stored at −80 °C in ~20% glycerol. All strains were grown in 50% De Man, Rogosa and Sharpe medium (MRS; 25.5 g/L), a selective culture medium for LAB strains (Sigma-Aldrich, St. Louis, USA). For plate solidification, 50% MRS medium was supplemented with 1.5% agar. Cultures were grown at 30 °C under static conditions.

### Experimental evolution

Experimental evolution was adopted by the bead-transfer model by Poltak and Cooper^17^. *L. lactis* and *L. mesenteroides* were grown as mono- and co-cultures, respectively, founded by five colonies of each species. The colonies were grown overnight, followed by OD_600_ adjustment to 0.05. For single-species cultures, 2 mL were added to a 24-well plate (Cellstar, Greiner Bio-One) containing three sterilized glass beads (~4 mm in diameter, VWR European). Co-cultures were prepared by mixing 1 mL of each species. After 24 h, a single bead was washed three times in phosphate-buffered saline solution (PBS) to remove loosely attached cells. After washing, the glass bead was transferred to a fresh 24-well plate containing 2 mL fresh 50% MRS broth and three clean marked glass beads (spot vs. no spot). The transfer procedure was repeated every 24 h. To verify purity during the transfer procedure, sterile beads in bacteria-free broth were transferred in parallel. At every transfer event, one colonized bead was stored in ~20% glycerol at −80 °C. Every third day, one bead was collected from each culture to enumerate cells and screen for emerging variations in colony morphology. The collected bead was washed three times in PBS and transferred to an Eppendorf tube containing 1 mL PBS. Cells adhered to the beads were detached by 5 min degassing and 5 min ultrasonic treatment at 40 kHz (Bransonic® 1510 Ultrasonic Cleaner, Branson). The detached cells were plated on 50% MRS agar plates complemented with 40 μg/mL Congo Direct Red 28 and 20 μg/mL Coomassie Blue G250 to distinguish colony morphologies. Plates were incubated at 30 °C for 48 h before enumeration and isolation.

The planktonic transfer model was conducted separately from the bead-transfer model. Overnight cultures of *L. lactis* and *L. mesenteroides* were OD_600_-adjusted to 0.05. The cultures were grown in 24-well plates (Cellstar, Greiner Bio-One) incubated under static conditions at 30 °C*. L. lactis* was grown as mono- and co-culture with *L. mesenteroides*, respectively. Every 24 h, the content of each well was homogenized by pipetting several times, and 2.5 µL was transferred to a new 24-well plate. Twenty parallel replicates were maintained for each culture type, founded by twenty colonies.

### Identification and verification of *L. lactis* colonies

To ensure that the isolated colonies from co-cultures were indeed *L. lactis*, the identity was confirmed by 16 S rRNA gene amplicon Sanger sequencing and lack of growth on plates supplemented with 100 µg/mL vancomycin, on which only *L. mesenteroides* is able to grow. Specifically, for Sanger sequencing (Macrogen, Europe), DNA was extracted from 2 mL overnight culture using FastDNA™ SPIN Kit for Soil (MP Biomedicals™) following the manufacturer’s instructions. DNA concentrations were measured using the Quant-iT™ High Sensitivity DNA Assay Kit (Life Technologies) before PCR. Phusion DNA polymerase (Thermo Scientific) was used to amplify the 16 S rRNA gene in 50 µL reactions with primers 27 F and 1492 R (Supplementary Table 4), annealing at 56 °C and 35 cycles. The PCR products were purified using the QIAquick PCR Purification Kit (QIAGEN™). Sequences were trimmed in CLC Genomics Workbench 10.0.01 using default settings. BLASTn (default settings)^21^ was used to identify *L. lactis* hits.

### Measurements of growth rate and culture yield

Growth curves were conducted in 50% MRS. Overnight cultures of isolates and the wildtype were diluted 500-fold in 50% MRS medium. For each culture, 300 µL was transferred to a 96-well cell culture plate (Sterile, F-bottom, with lid, Greiner bio-one, Cellstar) and grown under static conditions at 30 °C in a BioTek ELx808 (BioTek) using the Gen5 software (version 2.05) with OD_600_ measurements every 20 min. Growth curve data was analyzed based on the kinetic measurements using a modified version of the GrowthCurver package^23^, allowing for the occurrence of blank observations in the dataset. For culture yield data, samples were homogenized by pipetting post incubation and 250 µl was transferred to a new 96-well plate before measurement of optical density OD_600_. Four biological replicates were conducted to assess culture yield and growth rate (generation time).

### Crystal violet biofilm formation

A Calgary Biofilm Device^24^ was used to quantify biofilm formation under static conditions in 50% MRS. Overnight cultures of isolates and the wildtype were diluted 100-fold in 50% MRS medium in 96-well plates and covered with a lid with pegs extending into the wells (Nunc-TSP, Thermo-Scientific). After 48 h of incubation at 30 °C, the pegs were washed three times in PBS before biofilm was stained in 1% Crystal Violet for 20 min followed by a five-time washing step in PBS. The washed peg lids were placed in a 96-well plate containing 96% ethanol, and the absorbance of Crystal Violet was measured at 590 nm. Negative biofilm formation values, after background corrections, were adjusted to the lowest positive value measured.

### Quantitative PCR

Quantitative PCR (qPCR) was performed on the same biological replicates used to quantify culture yield and growth rate for all isolates. Cultures from the growth curve experiment were harvested in the early stationary phase. To account for potential biomass variation and precipitation among samples, cultures were homogenized with a pipet, and total biomass (yield) was estimated by measuring culture density at OD_600_. Bacterial DNA was extracted from 250 µL homogenized cultures using the PowerMag® Soil DNA Isolation Kit combined with the Eppendorf epMotion® 5075 platform. DNA extraction was performed according to the manufacturer’s recommendation with some minor modifications: (i) After bead beating, 600 µL supernatant was combined with 400 µL PowerMag® IRT solution. (ii) 800 µL supernatant was transferred to the MO BIO 2 mL Deep Well Plate before initiation of the DNA purification protocol. Eluted and purified DNA template was used for qPCR analysis with Brilliant III Ultra-Fast SYBR® Green QPCR Master Mix (Agilent). Each reaction mix contained 10 µL of 2x Brilliant III SYBR® Green QPCR Master Mix, 5–6 µL of PCR-grade water, 1 µL of universal eubacterial 16 S rRNA gene primer 341 F and 518 R, respectively (Supplementary Table 4) and 2–3 µL of DNA template. The reaction in the Lightcycler® 96 instrument (Roche) included a denaturation at 95 °C for 5 min followed by 45 cycles of 95 °C for 3 s, 60 °C for 30 s, followed by a melting curve with 95 °C for 60 s, 40 °C for 60 s, 65 °C for 1 s and 97 °C for 1 s. Each sample was running with a negative control, and obtained signals were compared to an in-house *E. coli* DNA standard to infer sample copy numbers.

### Plasmid construction

In order to generate a fluorescent signal in *L. lactis*, a derivative of pMG36C^25^ was constructed: PCR with primers sfGFP_XbaI_fw and sfGFP_PstI_rv (Supplementary Table 4) was performed with pSEUDO::Pusp45-*sfgfp*(Bs)^26^ as template, introducing XbaI and PstI restriction sites at the respective ends. The amplified fragments encoded superfolder GFP with codon usage optimized for *B. subtilis, sfgfp*(Bs), which previously has shown to yield the highest fluorescence intensities among different GFP variants tested in *L. lactis* subsp. *cremoris* MG1363^26^, and downstream terminators (*rrnB*, *rpsI*, and *tufA*). PCR product and backbone vector were digested with XbaI and PstI-HF in CutSmart buffer (New England Biolabs) and subsequently ligated with a T4 ligase (New England Biolabs) to generate pNAMA_P_32_-*sfgfp*(Bs)-Cm^R^. *E. coli* S17-1^27^ transformants harboring the plasmid were identified on LB agar supplemented with chloramphenicol. The plasmid was purified (Plasmid mini AX kit, A&A Biotechnology), and the construction was verified by PCR and subsequent Sanger sequencing (Macrogen, Europe) with primer pair pNAMA_veri (Supplementary Table 4).

### Transformation of pNAMA_P_32_-*sfgfp*(Bs)-Cm^R^

*L. lactis* was transformed as described by Holo & Nes^28^ with some modifications. Cells were grown to OD600 ≈ 0.5 in SOL1 (M17, 1.5% glycine, 0.25 M sucrose and 1% glucose) and harvested by centrifugation. The supernatant was discarded, the pellet washed in SOL2 wash buffer (M17, 10% glycerol and 0.5 M sucrose), and centrifugation was repeated. The final pellet was resuspended in SOL2 and mixed with pNAMA_P32-sfgfp(Bs)-CmR for electroporation in a 2 mm cuvette at 2.25 kV (Bio-rad GenePulser) and subsequent recovery in SGM17 (M17, 0.5% glucose, 0.2 M sucrose, 20 mM MgCl_2_ and 2 mM CaCl_2_) at 30 °C. Post recovery, cells were harvested by centrifugation, resuspended in 0.9% NaCl and plated on GSM17 (M17, 0.5 M sucrose, 0.5% glucose) agar plates supplemented with chloramphenicol to identify transformant colonies.

### Visualization by confocal laser scanning microscopy and image analysis

Planktonic cell cultures of *L. lactis* and *L. mesenteroides* were prepared as previously described with overnight cultivation followed by OD_600_ adjustment to 0.05. Autoclaved cover glass slides (18x18mm, Menzel-Gläser, Thermo Scientific) were placed in a 6-well plate, respectively, with support of 2–3 sterile glass beads (2 mm diameter) below the slide to give it a slight slant. For single-species cultures, 2 mL of OD-adjusted culture was added to the well. For mixed species cultures, 1 mL of each species was added. Well plates were incubated overnight under static conditions at 30 °C. Slides were washed before being visualized by confocal microscopy by submerging the slide in 2 mL PBS in a 12-well plate using a pair of tweezers. The wash procedure was performed three times per slide. Cells were visualized on the upward-facing side of the glass slide. A 488 nm laser was used for excitation and identification of *L. lactis* cells harboring pNAMA_P_32_-*sfgfp*(Bs)-Cm^R^ on the glass surface. 3D images were acquired in a 319.45 µm × 319.45 µm area (1024 × 1024 px × 0.312 µm/px) with *Z* range of 12 µm from various positions on each glass slide with an inverted CLSM instrument (Zeiss LSM 800, Carl Zeiss Inc., Germany) equipped with an EC Plan-Neofluar ×20/0.5 air objective and Axiocam 503 mono camera. Loading, preparation and quantification of images was conducted with RCon3D^29^ in RStudio^30^ using Otsu’s method for segmentation thresholding^31^. To visualize the localization of *L. lactis* in co-culture, the mixed culture was stained with DAPI (final conc. 25 µM) for 20 min, before slides were washed. A 488 nm laser was used for excitation of sfGFP, while a 405 nm laser was used for excitation of DAPI. Images were acquired with the same ×20 objective as above, and with a Plan-Achromat ×63/1.4 oil DIC M27 objective. For the latter, images were acquired in an area of 77.248 µm × 77.248 µm area (2272 × 2272 px × 0.034 µm/px) with application of airyscan detector mode. These settings were also applied to image the entanglement of the S-morphotype and lack of such in the ancestor with a 488 nm laser, after 20 min of staining with Syto16 (ThermoFisher) (final conc. 25 µM).

### Quantification of number of cells per chain

To quantify the number of cells per chain, cells were grown for 24 h and subsequently gently diluted 1:10 in 0.9% NaCl solution on a microscope slide before a #1.5 cover glass was placed on top. A minimum of 12 chains were counted, representing 6 different areas of each sample, using a BH2 series light microscope (Olympus, Japan) equipped with a ×100/1.3 oil objective. The experiment was repeated three independent times.

### Statistical analysis

All statistical analyses were performed in the R environment using the tidyverse^32^ version 1.1.3 and foreach version 1.5.1 packages for data management and visualization. For comparison of temporal CFU/mL dynamics we used Welch two sample t-tests comparing each day separately (three technical replicates were averaged for each replicate). Data from lineage A at day 13 and lineage C day 7 were excluded from the co-culture dynamics analysis due to technical issues. Comparison of relative abundance at day 16 was done using Wilcoxon rank-sum test (three technical replicates were averaged for each replicate). To compare the characteristics of the evolved isolates and the ancestors, the log2 ratio of biofilm formation, generation time, final pH, copy numbers and culture yield was designated as response variable, respectively, in a mixed-effect linear model, with application of the *lmer* function from the package *lme4*^33^, referred to as MELM. Fixed dummy variables included culture type, morphotype and the interaction between the two. The evolution lineage (*N* = 5) was included as random intercept. Observations that had a Cook’s distance greater than four times the mean were classified as influential and removed prior to the MELM analysis. In total, for culture yield, generation time and copy number, the average of biological replicates was regarded as independent observations (*N* = 79), while for biofilm formation and acidification of media (pH), the average of three biological replicates was regarded as independent observations (*N* = 85, *N* = 12, respectively). *P* values were FDR adjusted^34^ separately for each of the respective models.

### Mutation analysis

Whole-genome sequences from ancestral strains and variants were obtained using Illumina MiSeq platform by sequencing of individual Nextera XT libraries obtained from each isolate DNA extraction. Sequencing was performed in paired-end mode using Illumina MiSeq v3 reagents kit (2 × 300 cycles). Raw sequence reads were quality trimmed (Phred score: 20) and Nextera adapter sequences were removed with the Perl wrapper tool Trim Galore v0.6.7 (https://github.com/FelixKrueger/TrimGalore) using default settings (July 2021). Trim Galore makes use of cutadapt^35^ and FastQC (https://www.bioinformatics.babraham.ac.uk/projects/fastqc/). To investigate mutations among the *L. lactis* ancestor and evolved variants, paired and unpaired reads were mapped to the reference strain *L. lactis* strain FDAARGOS_865^36^ using the computational pipeline breseq (version: 0.35.5) with default settings^37^.

### Reporting summary

Further information on research design is available in the Nature Research Reporting Summary.

## Supplementary information

Supplemental material

## Data Availability

The raw Illumina MiSeq sequence reads obtained in this study have been deposited in the European Nucleotide Archive (ENA) under study number PRJEB42737. All CLSM images and specific data are available upon request.

## Code availability

Data and scripts can be obtained at https://github.com/nh91/Co-Perform.
