# Supplementary material for: Biofilm cultivation facilitates coexistence and adaptive evolution in an industrial bacterial community
Source: NPJ Biofilms Microbiomes. 2022 Jul 20;8:59. doi: 10.1038/s41522-022-00323-x (PMC9300721; doi:10.1038/s41522-022-00323-x)
Supplement: Supplementary file 1 — Supplemental material [file 41522_2022_323_MOESM1_ESM.pdf]

Supplementary information

Supplementary Table 1: Generation times of *Lactococcus lactis* and *Leuconostoc mesenteroides*

	Species	*g* (hours per generation)		Total *g* (14 days)
**Bead-transfer model**		**Bead**	**Suspension**	**Bead**
Mono-culture	*L. lactis*	3.1	3.2	108
	L. mesenteroides	3.96	2.26	85
Co-culture	*L. lactis*	3.1	3.2	108
	L. mesenteroides	3.96	2.26	85
**Planktonic-transfer model**				
Mono-culture	*L. lactis*		3.2	105
Co-culture	*L. lactis*		3.2	105
	L. mesenteroides		2.25	149

The generation time was estimated by adjusting OD600 of an overnight culture to 0.05 in a 24-well plate containing 4 glass beads. Cells were grown statically at 30°C.
After 24h of growth, beads were washed in PBS and moved to a new well with fresh medium (50% MRS) and new beads (marked with dot).
New beads were collected at T=3h and T=24h and sonicated to remove adhering cells.
Detached cells and a sample from the suspension were plated on 50% MRS agar plates and incubated for 48 hours at 30°C.
Based on cell number enumeration, generation time was calculated as log2(CFU24h / CFU3h) and the hours per generation was then adjusted for the 21h of time difference.
Values are averages from 5 biological replicates.

The generation time in planktonic culture was measured with a CFU count from the culture at t=3h and t=24h and calculated as above.
Values are averages from 20 biological replicates.
We suspect the relative high generation time of *L. mesenteroides* on bead reflects dispersal.
If cells did detach/disperse within the time frame, it would artificially increase the generation time, as the calculation would be based on a lower number of cells, than had been in the biofilm.

Supplementary Table 2: Detailed information on BreSeq prediction of mutations

Predicted mutations																										
position	mutation	S21	S22	S23	S24	S25	S26	S27	S28	S29	S30	S31	S32	S33	S34	S35	S36	S37	S38	S39	S40	S41	S42	annotation	gene	description
74.239	G→C						100%										100%	100%				100%		F200L (TTC→TTG)	*I6G22_RS00675* ←	APC family permease
103.059	$(A)_{10\to9}$							100%			100%													intergenic (-238/+27)	*I6G22_RS00805* ← / ← *rpmA*	Nramp family divalent metal transporter/50S ribosomal protein L27
184.158	C→A								?	?												100%		pseudogene (384/3270 nt)	*I6G22_RS01325* ←	hypothetical protein
201.295	T→G				100%	100%				100%						100%								K107Q (AAA→CAA)	*trpD* ←	anthranilate phosphoribosyltransferase
418.982	G→T																		100%					P696H (CCC→CAC)	*I6G22_RS02370* ←	DNA translocase FtsK
448.442	A→C							100%			100%													T79T (ACT→ACG)	*I6G22_RS02490* ←	alpha-amylase
459.906	C→A													?							100%			E67* (GAG→TAG)	*I6G22_RS02540* ←	helix-turn-helix transcriptional regulator
459.909	+T											100%												coding (196/207 nt)	*I6G22_RS02540* ←	helix-turn-helix transcriptional regulator
459.980	G→T										100%													A42D (GCT→GAT)	*I6G22_RS02540* ←	helix-turn-helix transcriptional regulator
460.083	C→A																						100%	D8Y (GAT→TAT)	*I6G22_RS02540* ←	helix-turn-helix transcriptional regulator
position	mutation	S21	S22	S23	S24	S25	S26	S27	S28	S29	S30	S31	S32	S33	S34	S35	S36	S37	S38	S39	S40	S41	S42	annotation	gene	description
484.790	G→A	100%	100%						100%			100%	100%	100%	100%				100%	100%			100%	G208D (GGT→GAT)	*I6G22_RS02675* →	PTS mannose/fructose/sorbose transporter family subunit IID
558.881	G→A													100%										A439V (GCA→GTA)	*I6G22_RS03075* ←	F0F1 ATP synthase subunit alpha
618.320	G→T			100%																				S53* (TCA→TAA)	*I6G22_RS03340* ←	DNA/RNA non-specific endonuclease
653.473	C→T			100%																				intergenic (-491/+11)	*infC* ← / ← *I6G22_RS03530*	translation initiation factor IF-3/IS6 family transposase
698.291	G→C																		100%					intergenic (-166/+484)	*I6G22_RS03750* ← / ← *I6G22_RS03755*	tRNA-OTHER/C40 family peptidase
703.500	G→T				100%																			L565I (CTT→ATT)	*I6G22_RS03790* ←	phage tail protein
891.509	2 bp→CA					100%																		coding (821-822/1683 nt)	*I6G22_RS04755* →	ribonuclease J
952.166	C→T													100%										G206D (GGT→GAT)	*hflX* ←	GTPase HflX
1.070.063	C→T				100%	100%	100%			100%						100%	100%	100%				100%		R231K (AGA→AAA)	*I6G22_RS05660* ←	16S rRNA (uracil(1498)-N(3))-methyltransferase
1.083.236	A→G								100%															intergenic (-100/+95)	*I6G22_RS05745* ← / ← *I6G22_RS05750*	hypothetical protein/hypothetical protein
position	mutation	S21	S22	S23	S24	S25	S26	S27	S28	S29	S30	S31	S32	S33	S34	S35	S36	S37	S38	S39	S40	S41	S42	annotation	gene	description
1.141.824	C→T																		100%					G270E (GGA→GAA)	*I6G22_RS06110* ←	transcription antiterminator
1.210.950	G→T																	100%						S296Y (TCT→TAT)	*I6G22_RS06485* ←	DNA primase
1.314.198	+TC			100%				100%			100%										100%			coding (70/1026 nt)	*I6G22_RS07000* ←	lactonase family protein
1.394.479	C→T																			100%				T98I (ACT→ATT)	*I6G22_RS07470* →	glutamate--tRNA ligase
1.548.839	G→A														100%									intergenic (+356/-205)	*I6G22_RS08265* → / → *thrS*	ribonucleotide reductase/threonine--tRNA ligase
1.634.101	Δ1 bp		100%																					coding (439/2301 nt)	*I6G22_RS08825* →	glycoside hydrolase family 65 protein
1.679.398	A→G														100%									D120G (GAC→GGC)	*I6G22_RS09110* →	trigger factor
1.909.821	G→A																100%							E80K (GAA→AAA)	*I6G22_RS10245* →	acetyl-CoA carboxylase biotin carboxyl carrier protein
1.961.207	G→T			100%																				A28S (GCT→TCT)	*sdaAB* →	L-serine ammonia-lyase, iron-sulfur-dependent subunit beta
2.144.431	G→A				100%																			D79N (GAT→AAT)	*I6G22_RS11315* →	hypothetical protein
position	mutation	S21	S22	S23	S24	S25	S26	S27	S28	S29	S30	S31	S32	S33	S34	S35	S36	S37	S38	S39	S40	S41	S42	annotation	gene	description
2.214.489	G→T						100%																	L930L (CTG→CTT)	*carB* →	carbamoyl-phosphate synthase large subunit
2.311.011	C→G				100%	100%	100%			100%						100%	100%	100%				100%		P20R (CCC→CGC)	*I6G22_RS12180* →	hypothetical protein
2.365.130	T→G							100%			100%													intergenic (+86/-225)	*I6G22_RS12385* → / → *I6G22_RS12390*	nucleotide sugar dehydrogenase/helix-turn-helix transcriptional regulator

Supplementary Table 3: Bacterial strains in this study

Species	Strain	Characteristics	Lineage	Sampling day	Source
L. mesenteroides subsp. *Mesenteroides*	DSM-20343	Wildtype	-	-	DSMZ
L. lactis subsp. *Lactis*	DSM-20481	Wildtype	-	-	DSMZ
	S21/Ancestor1/MBLa1				
L. lactis subsp. *Lactis*	DSM-20481	Wildtype	-	-	DSMZ
	S22/Ancestor2/MBLa2				
L. lactis subsp. *Lactis*	DSM-20481	Wildtype harboring pNAMA_P32-sfgfp(Bs)-CmR	-	-	This study
	MBLa102				
L. lactis subsp. *Lactis*	S23/XA.2-l/MBLa11	Single-evolved	A	14	This study
L. lactis subsp. *Lactis*	S24/XB.1-l/MBLa12	Single-evolved	B	14	This study
L. lactis subsp. *Lactis*	S25/XB.2-l/MBLa13	Single-evolved	B	14	This study
L. lactis subsp. *Lactis*	S26/XC.3-l/MBLa14	Single-evolved	C	14	This study
L. lactis subsp. *Lactis*	S27/XD.1-l/MBLa15	Single-evolved	D	14	This study
L. lactis subsp. *Lactis*	S28/XE.1-l/MBLa16	Single-evolved	E	14	This study
L. lactis subsp. *Lactis*	S29/XB.5-m/MBLa17	Single-evolved	B	14	This study
L. lactis subsp. *Lactis*	S30/XD.5-m/MBLa18	Single-evolved	D	14	This study
L. lactis subsp. *Lactis*	S31/XE.4-m/MBLa19	Single-evolved	E	14	This study
L. lactis subsp. *Lactis*	S32/XYE.2-l/MBLa20	Co-evolved	E	14	This study
L. lactis subsp. *Lactis*	S33/XYA.4-l/MBLa21	Co-evolved	A	14	This study
L. lactis subsp. *Lactis*	S34/XYA.5-l/MBLa22	Co-evolved	A	14	This study
L. lactis subsp. *Lactis*	S35/XY.B4-l/MBLa23	Co-evolved	B	14	This study
L. lactis subsp. *Lactis*	S36/XYC.1-l/MBLa24	Co-evolved	C	14	This study
L. lactis subsp. *Lactis*	S37/XYC.4-l/MBLa25	Co-evolved	C	14	This study
L. lactis subsp. *Lactis*	S38/XYE.1-l/MBLa26	Co-evolved	E	14	This study
L. lactis subsp. *Lactis*	S39/XYE.3-l/MBLa27	Co-evolved	E	14	This study
L. lactis subsp. *Lactis*	S40/XYA.3-m/MBLa28	Co-evolved	A	14	This study
L. lactis subsp. *Lactis*	S41/XYC.4-m/MBLa29	Co-evolved	C	14	This study

L. lactis subsp. *Lactis*	S42/XYE.4-m/MBLa30	Co-evolved	E	14	This study
L. lactis subsp. *Lactis*	DSM-20481<br>S183/Ancestor3/MBLa3	Wildtype	-	-	DSMZ
L. lactis subsp. *Lactis*	DSM-20481<br>S184/Ancestor4/MBLa4	Wildtype	-	-	DSMZ
L. lactis subsp. *Lactis*	DSM-20481<br>S185/Ancestor5/MBLa5	Wildtype	-	-	DSMZ
L. lactis subsp. *Lactis*	DSM-20481<br>S186/Ancestor6/MBLa6	Wildtype	-	-	DSMZ
L. lactis subsp. *Lactis*	DSM-20481<br>S187/Ancestor7/MBLa7	Wildtype	-	-	DSMZ
L. lactis subsp. *Lactis*	DSM-20481<br>S188/Ancestor8/MBLa8	Wildtype	-	-	DSMZ
L. lactis subsp. *Lactis*	DSM-20481<br>S189/Ancestor9/MBLa9	Wildtype	-	-	DSMZ
L. lactis subsp. *Lactis*	XI.a1/MBLa31	Single-evolved	A	16	This study
L. lactis subsp. *Lactis*	XY.b2/MBLa32	Co-evolved	B	16	This study
L. lactis subsp. *Lactis*	XY.e/MBLa33	Co-evolved	E	16	This study
E. coli	S17-1	*thi pro hsd*R *hsdM*$^{+}$ *recA* RP4-2-Tc::Mu-Km::Tn7	-	-	[1]

Supplementary Table 4: Plasmids and oligonucleotides in this study

Plasmid	Characteristics	Reference
pMG36C	Cm^R, P_{32} promoter	[2]
pSEUDO::Pusp45-*sfgfp*(Bs)	ery^R, *sfgfp*(Bs), P_{ups45} promoter	[3]
pNAMA_P_{32}-*sfgfp*(Bs)-Cm^R	pMG36C-derivate, Cm^R, *sfgfp*(Bs), P_{32} promoter	This study

Primer	Sequence (5'-3')
27F	AGAGTTTGATCMTGGCTCAG
341F	CCTACGGGAGGCAGCAG
581R	ATTACCGCGGCTGG
1492R	CGGTTACCTTGTTACGACTT
sfGFP_XbaI_fw	ACG<u>TCTAGA</u>CGAGTACTGATTAACTAATAAGGAGG
sfGFP_PstI_rv	TTA<u>CTGCAG</u>AGCGCTATCAAAAGAATCTTGC
pNAMA_veri_fw	CAGCTCCAGATCGATTCA
pNAMA_veri_rv	GAAGTCAGCTGCCTAAGC

Abbreviations: Cm^R: Chloramphenicol resistance, ery^R: Erythromycin resistance

Supplementary Figure 1: Growth characteristics for *Lactococcus lactis* and *Leuconostoc mesenteroides* ancestors.

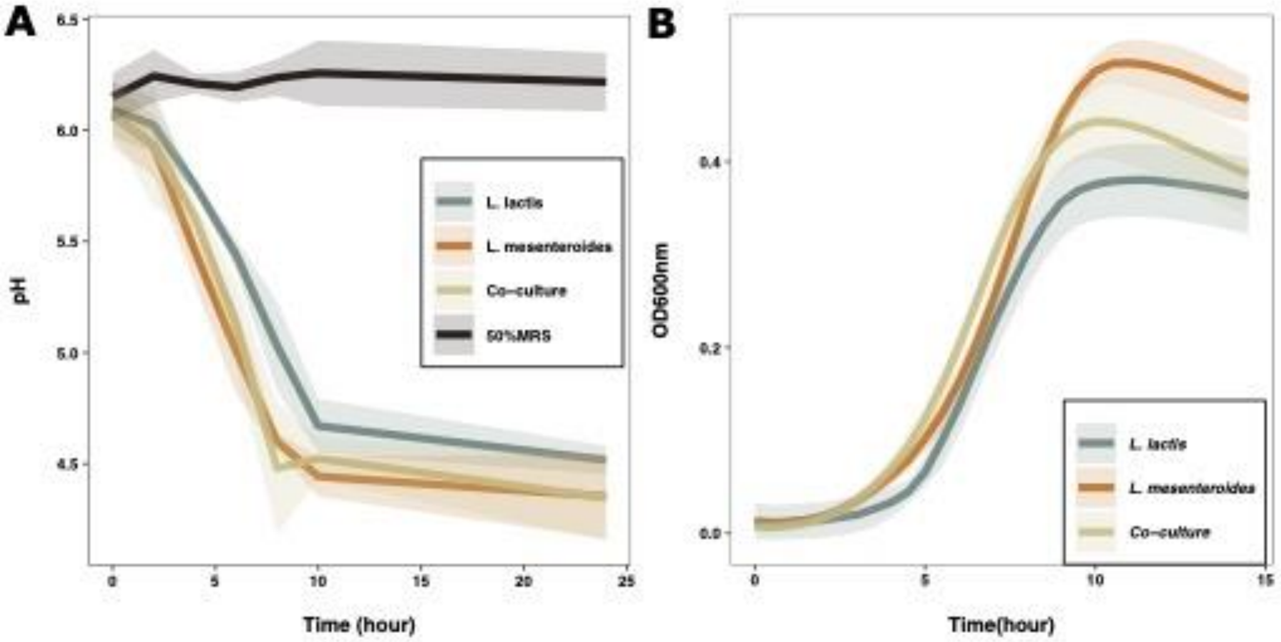

A) pH profiles of *L. lactis* and *L. mesenteroides* in mono- and co-cultures. pH was measured every second hour for 12 hours, with a final measurement after 24 hours. The cultures were grown statically in 50% MRS broth at 30 °C. Means and 95% confidence intervals are shown as solid lines and ribbons, respectively. Data is based on 3 biological replicates ($N = 3$). **B)** Growth curves of *L. lactis* and *L. mesenteroides*. Species were grown statically at 30 °C in 50% MRS broth. Growth curves were measured as absorbances (OD_{600}) in a 96-well plate reader. Measurements were performed every 20 min for 16 hours. Means and 95% confidence intervals are shown as solid lines and ribbons, respectively. Data is based on 3 biological replicates ($N = 3$).

Supplementary Figure 2: Colony morphology of ancestors and evolved *Lactococcus lactis* variants.

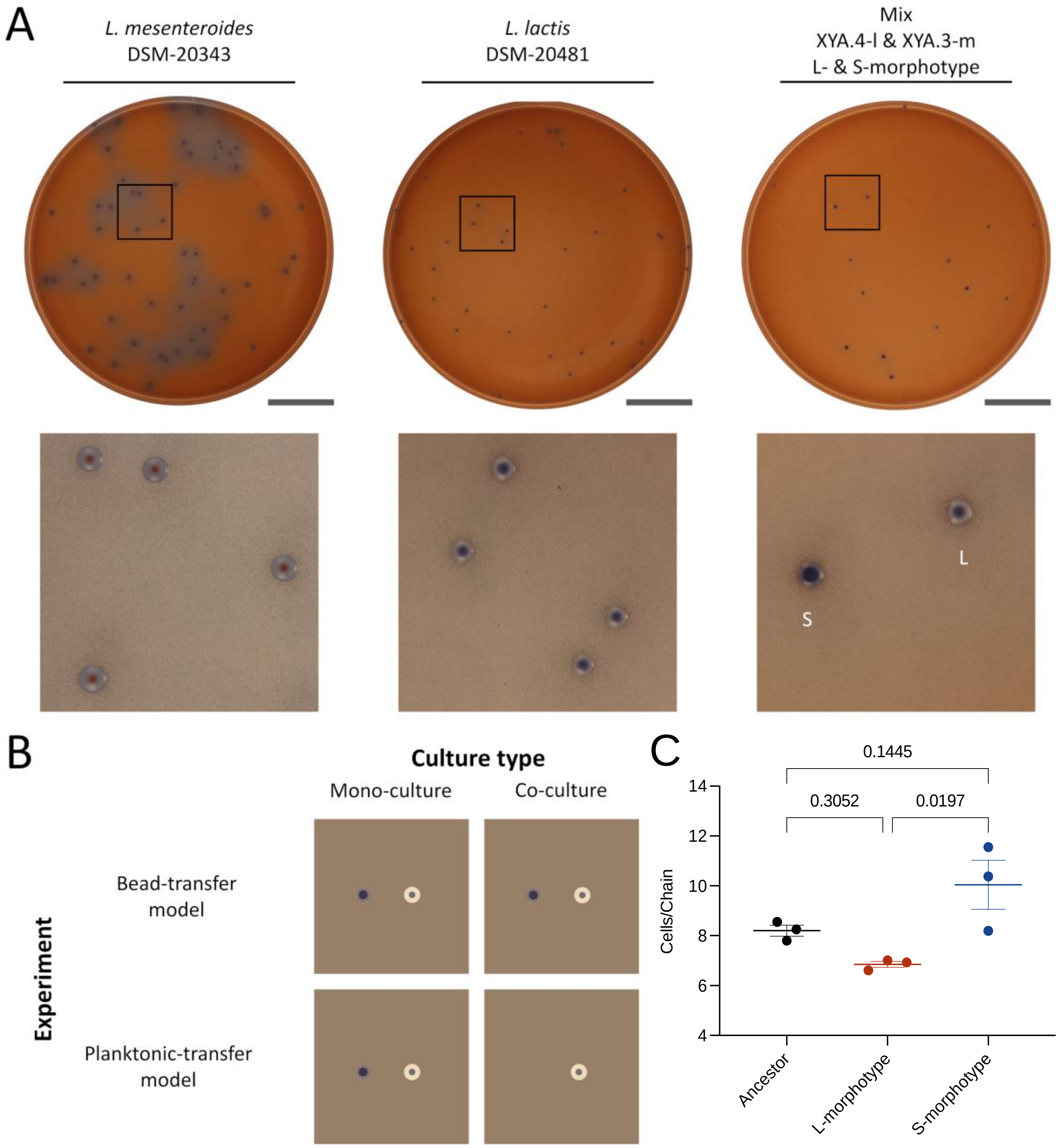

A) Images of colony morphologies of *L. mesenteroides* and *L. lactis* ancestor and *L. lactis* evolved variants L- and S-Morphotype. The colonies were grown on 50% MRS agar plates containing 40 µg/mL Direct Red 28 and 20 µg/mL Coomassie Blue G250. The scale bar represents 2cm **B)** Schematic overview of *L. lactis* colony morphologies observed in the two evolution experiments (bead-transfer model and planktonic-transfer) in the two different cultivation types (mono-culture and co-culture with *L. mesenteroides*). S- and L-morphotype were observed in all cultures except from co-cultures in the planktonic-transfer model, where only the L-morphotype was recognized. **C)** The average chain length varies between the respective morphotypes (P = 0.0197, one-way ANOVA and Tukey's multiple comparison test). The number of cells was quantified in a subset of isolates (Two ancestor, nine L-morphotype and four S-morphotype). At least 12 chains were counted from each isolate, and the experiment was repeated three times.

Supplementary Figure 3: Overnight cultures of *Lactococcus lactis* ancestor and bead-transfer evolved variants.

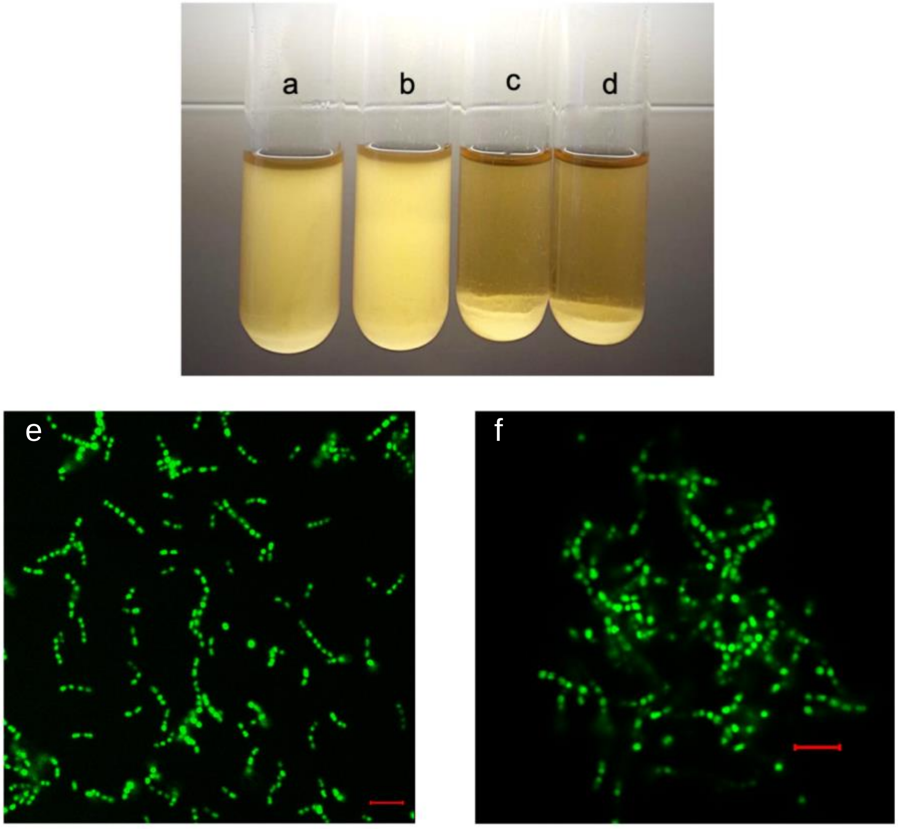

A) Planktonic growth behavior of ancestral *L. lactis*. **B)** Planktonic growth behavior of an L-morphotype variant (Xl.A1/MBLa31) isolated from a bead-transfer mono-culture (lineage A) at day 16. **C)** Clumping growth behavior of an S-morphotype variant (XY.B2/MBLa32) isolated from a bead-transfer co-culture (lineage B) at day 16. **D)** Clumping growth behavior of an S-morphotype variant (XY.E/MBLa33) isolated from a bead-transfer co-culture (lineage E) at day 16. Cultures were inoculated with a single colony from 50% MRS agar plates and grown in 50% MRS broth statically at 30 °C. Photos were acquired 20 hours after inoculation. **E)** Visualization of the ancestral *L. lactis* at 63x magnification from liquid culture showed high variation in numbers of cells per chain and a mixed distribution. **F)** Visualization of the S-morphotype at 63x magnification reveal a high level of chain entanglement, where cells tended to create a mesh. Scale bars represent 5μm.

Supplementary Figure 4: Co-cultivation increased the number of *Lactococcus lactis* cells present in the early stationary phase when grown in mono-culture.

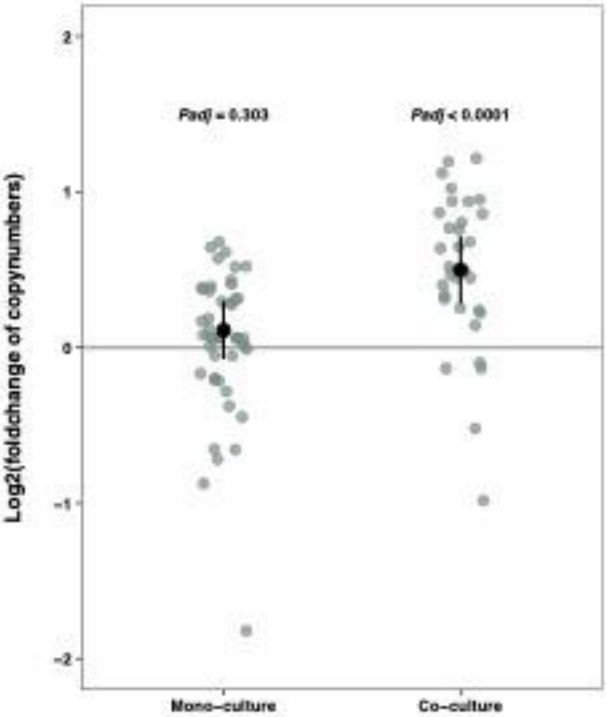

Comparative analyses of *L. lactis* isolates emerging from mono- and co-cultivation in the bead-transfer model, respectively, reveal that co-cultivation enabled unique evolution of isolates with significantly increased number of cells (assessed by qPCR) when reaching early stationary phase in a mono-culture growth assay ($P < 0.0001$, MELM). Growth of isolates and ancestor cells were monitored as optical density (OD_{600}) in a microtiter plate until reaching the early stationary phase, where all cultures were sampled, and cell numbers were estimated using qPCR. Differences between mono- and co-culture copy numbers as compared to the ancestor were estimated with a mixed-effects linear model. The enhanced number of cells was unique for the co-culture isolates. One observation represents the average of four biological replicates (N = 79). Black symbols represent estimated population mean by MELM, error bars represent 95% confidence intervals and green symbols represents observations. *P* values are adjusted using FDR.

Supplementary Figure 5: Mixed-effects linear models variables for culture yield, generation time and biofilm formation for evolved *Lactococcus lactis* isolates.

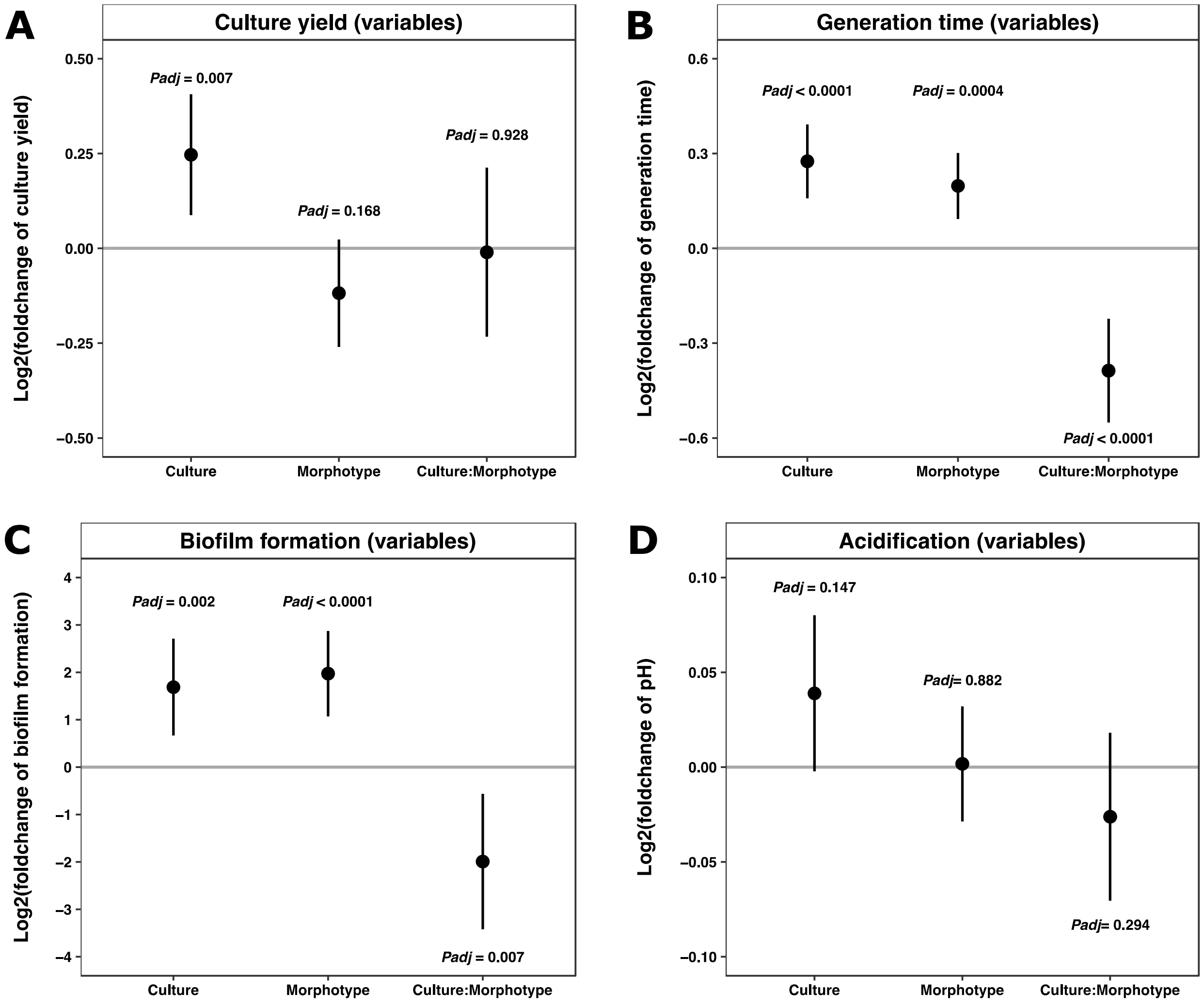

A) Variables originating from the comparative analyses of *L. lactis* isolates emerging from mono- and co-cultivation using a mixed-effects linear model (MELM). The type of cultivation (Culture) has a significant output, meaning that isolates originating from mono- and co-cultures have significantly different culture yields. Culture yield was estimated as the maximum culture yield of the culture from a logistic equation from the R-package Growthcurver. Culture yield of all mono- and co-culture isolates were normalized to the culture yield of the ancestor. Data are based on the average of four biological replicates (N = 79). **B)** Variables originating from the comparative analyses of generation time of mono- and co-culture isolates using a MELM. The culture type (Culture) and morphotype type (Morphotype; L- or S-morphotype) were significantly different between mono- and co-culture isolates. Also the interaction term between the two variables (Culture:Morphotype) was significantly different. Thus, the effect of the culture type on the generation time of the isolates depended on the morphotype of the variant. Generation times were estimated from growth-monitored overnight cultures of ancestor and mono- and co-culture isolates of *L. lactis*, based on OD_{600} measurements and fitted growth curves from the GrowthCurver package. Generation times of all mono- and co-culture isolates were normalized to the growth rate of the ancestor. Data is based on the average of four biological replicates (N = 79) **C)** Variables originating from the comparative analyses of mono- and co-culture isolates biofilm formation using a MELM. The culture type (Culture) and morphotype type (Morphotype; L- or S-morphotype) were significantly different

between mono- and co-culture isolates. Also the interaction term between the two variables (Culture:Morphotype) was significant, thus the effect of the culture type on the biofilm formation capability of the isolates depended on the morphotype of the isolate. Biofilm formation was estimated after 48 hours of static growth in the Calgary device using Crystal Violet (OD_{590}). Negative biofilm formation values, after background correction, were adjusted to the lowest positive value measured. Data is based on the average of three biological replicates (N = 85). **D)** Variables originating from the comparative analysis of mono- and co-culture isolates final pH using a MELM. None of the parameters tested were significantly different from the ancestor. Data are based on the average of three biological replicates (*N* = 12). Graphs depict means, 95% confidence intervals and adjusted *P* values using FDR.

References

1. Simon R, Priefer U, Pühler A. A Broad Host Range Mobilization System for In Vivo Genetic Engineering: Transposon Mutagenesis in Gram Negative Bacteria. *Nat Biotechnol* [Internet]. 1(9), 784–791 (1983). Available from: https://www.nature.com/articles/nbt1183-784.
2. Van De Guchte M, Van Der Vossen JMBM, Kok J, Venema G. Construction of a lactococcal expression vector: Expression of hen egg white lysozyme in Lactococcus lactis subsp. lactis. *Appl. Environ. Microbiol.* 55(1), 224–228 (1989).
3. Overkamp W, Beilharz K, Weme RDO, *et al.* Benchmarking various green fluorescent protein variants in Bacillus subtilis, Streptococcus pneumoniae, and Lactococcus lactis for live cell imaging. *Appl. Environ. Microbiol.* 79(20), 6481–6490 (2013).
